# Application of improved and optimized fuzzy neural network in classification evaluation of top coal cavability

**DOI:** 10.1038/s41598-021-98630-4

**Published:** 2021-09-28

**Authors:** Meng Wang, Caiwang Tai, Qiaofeng Zhang, Zongwei Yang, Jiazheng Li, Kejun Shen

**Affiliations:** College of Mining Engineering, Liaoning Technical University, Fuxin, 123000 China

**Keywords:** Civil engineering, Energy infrastructure

## Abstract

Longwall top coal caving technology is one of the main methods of thick coal seam mining in China, and the classification evaluation of top coal cavability in longwall top coal caving working face is of great significance for improving coal recovery. However, the empirical or numerical simulation method currently used to evaluate the top coal cavability has high cost and low-efficiency problems. Therefore, in order to improve the evaluation efficiency and reduce evaluation the cost of top coal cavability, according to the characteristics of classification evaluation of top coal cavability, this paper improved and optimized the fuzzy neural network developed by Nauck and Kruse and establishes the fuzzy neural network prediction model for classification evaluation of top coal cavability. At the same time, in order to ensure that the optimized and improved fuzzy neural network has the ability of global approximation that a neural network should have, its global approximation is verified. Then use the data in the database of published papers from CNKI as sample data to train, verify and test the established fuzzy neural network model. After that, the tested model is applied to the classification evaluation of the top coal cavability in 61,107 longwall top coal caving working face in Liuwan Coal Mine. The final evaluation result is that the top coal cavability grade of the 61,107 longwall top coal caving working face in Liuwan Coal Mine is grade II, consistent with the engineering practice.

## Introduction

Longwall top coal caving mining is a special type of mining method, which is usually used to mine coal seams with a thickness of more than 4.5 m^1^. Because longwall top coal caving mining has apparent advantages such as low excavation rate, high efficiency, strong adaptability, and easy to achieve high yield^2^, China introduced this technology from abroad in the 1880s. After the technology entered China, this technology has become China's primary technology for mining thick coal seams through continuous development and innovation^3^. At present, there are more than 200 working faces in China using longwall top coal caving mining technology to mine thick coal seams. Suppose the top coal cavability can be evaluated scientifically and accurately before adopting this technology to mine thick coal seams. And then, based on the results of the classification evaluation of top coal cavability, targeted mining design, and preventive measures should be taken. In that case, it will provide a strong guarantee for the success of longwall top coal caving mining. Because the top coal cavability is one of the most important parameters to evaluate the applicability of longwall top coal caving mining. It is also an important method to reduce technical risk from the feasibility analysis stage to the design stage in mining design^4^.

However, in coal production at home and abroad, empirical and numerical simulation methods are widely used to evaluate top coal cavability^5^, but both have shortcomings. For example, the empirical method will be difficult to evaluate the top coal cavability accurately when there is no precedent to follow. Apart from that, with the increasing size of the model, the requirements for computers and software are also higher and higher, and the time required for model calculation is longer and longer, which makes the efficiency of using numerical simulation method to evaluate the top coal cavability not high. Therefore, it is necessary to find a more simple method to evaluate the top coal cavability.

However, under the era background that advanced artificial intelligence algorithms such as ant colony clustering algorithm^6^, expert system^7^, and neural network^8^ are gradually applied to coal production, experts and scholars at home and abroad also begin to try to apply these advanced algorithms to the classification evaluation of top coal cavability. For example, Sodjad Mohammadi and Mohammad Ataei et al.^9^ used fuzzy multi-criteria decision-making methods to establish a classification system for evaluating the cavability of the direct roof of a coal seam and used it to evaluate 12 working faces. Shi Yongkui and Li Pengrui et al.^10^ used Bayesian theory and rough set theory to establish a Bayesian classifier model for evaluating and predicting coal roof cavability and used the model to predict eight groups of samples to be tested, with an accuracy of 100%. Moreover, Kazem Oraee and Mehrshad Rostami^11^ established a fuzzy system for quantitative analysis of roof cavability in longwall top coal caving working face by using the fuzzy logic algorithm and applied the model to the evaluation and prediction of roof cavability in Tabas Palward Coal Mine in Palward District, Yazd Province, Iran. In the application, the predicted results of the model are consistent with the field-measured results. However, the models established by the above experts and scholars meet the engineering needs to a certain extent, and there are certain defects in varying degrees, such as complex modeling process, poor model portability, and too few samples in the modeling process which makes its universal applicability questionable. In addition, they did not realize that the evaluation of top coal cavability is a problem of fuzziness, complexity, and uncertainty, in essence, and this fuzziness and uncertainty is different from the randomness in probability theory, but the uncertainty in people's subjective understanding, that is, the uncertainty in people's definition or concept description of events in the language sense^12^. However, the fuzzy theory was born to solve this problem, and now the fuzzy theory is also being widely used to deal with this problem.

Therefore, based on clearly realizing that the classification evaluation of top coal cavability problem is essentially a problem with fuzziness, complexity, and uncertainty, the fuzzy neural network formed by the organic combination of fuzzy method and neural network is used to establish a model that can be applied to the classification evaluation of top coal cavability. From which to make the classification evaluation of top coal cavability model have good portability and universal adaptability. Because fuzzy nerves use neural networks to find the parameters related to the fuzzy system through learning given data. These parameters include fuzzy sets, fuzzy rules, fuzzy membership functions, and etcetera. In addition, fuzzy neural networks can be established with or without prior knowledge of fuzzy rules and can keep the underlying fuzzy system unchanged in the learning process^13^. Therefore, the fuzzy neural network has the characteristics of both the fuzzy method and neural network. The fuzzy method can effectively represent subjective, fuzzy language and inaccurate data and information^14^; The neural network has high fault tolerance and fast convergence characteristics. In short, it can be considered that as long as having data, can use a fuzzy neural network to find a fuzzy neuro system from it^13^. However, according to the current reports on top coal cavability, it is difficult to ensure that complete fuzzy rules and fuzzy sets can be established, so applying a fuzzy neural network is the best method.

Nevertheless, the general fuzzy neural network has disadvantages, such as low prediction accuracy and extended training time^15^. Therefore, a fuzzy neural network for data analysis developed by Nauck and Kruse^16^ is used as the reference form of the fuzzy neural network structure, and an improved and optimized fuzzy neural network prediction model is established based on the structure, which is applied to the classification evaluation of top coal cavability. It is hoped to provide another efficient, scientific and accurate method for top coal cavability evaluation and improve the efficiency of top coal cavability evaluation.

## Propose an improved fuzzy neural network and its global approximation verification

### Propose an improved fuzzy neural network

**Figure 1:**
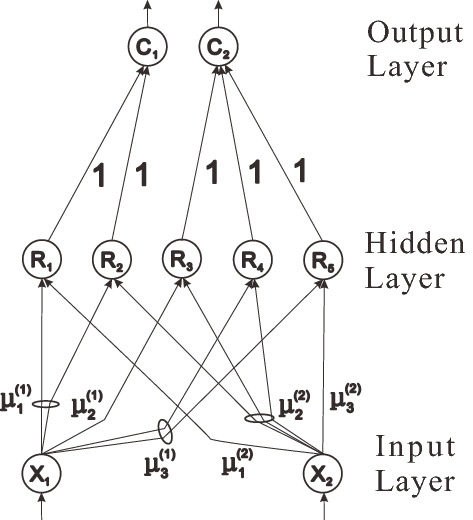
Schematic diagram of a neural network composed of two inputs, five rules, and two outputs developed by Nauck and Krus.

**Figure 2:**
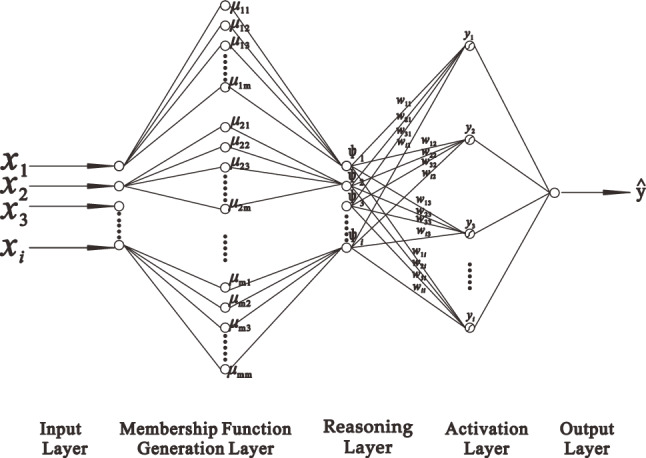
Optimized and improved neural network structure.

The fuzzy neural network developed by Nauck and Kruse is a fuzzy neural network composed of the input layer, hidden layer or rule layer, and output layer, in which the first layer fuzzies the clear input parameters, the second layer is the precondition of ruleset, the third layer performs defuzzification operations, as shown in Fig. 1. This paper improves the network under this framework and changes the network into a fuzzy neural network composed of input layer, membership generation layer, reasoning layer, activation layer, and output layer, as shown in Fig. 2. The details of each layer are as follows:

*Layer 1* This layer is the input layer, and clear independent variable values are input.

*Layer 2* This layer is the membership generation layer. The clear independent variable value passed in from layer 1 will be fuzzified by the membership function set in this layer. At this time, the clear variable value will be transformed into the corresponding membership fuzzy value and complete the fuzzy processing.

*Layer 3* This layer is the reasoning layer. The fuzzy value of membership passed in from layer 2 is conjunct calculations, and the corresponding fuzzy rules are formed. Therefore, each neuron in this layer is a fuzzy rule. In addition, the output of the neurons in this layer is the activation strength of the association rules.

*Layer 4* This layer is the activation layer, which combines the activation intensity of all association rules passed in from layer 3 to form the estimation of class to achieve the effect of defuzzification, thereby obtaining a clear value. The activation function processes the clear value to obtain the label code of the corresponding prediction category.

*Layer 5* This layer is the output layer. In this layer, the final output value is finally obtained by inverse coding the coding of the prediction category passed in from layer 4 (In classification problems, the target value is generally performed one-hot coded during preprocessing).

The improved fuzzy neural network membership function adopts the Gaussian membership function because some studies^17,18^ show that taking the Gaussian membership function as the membership function of the fuzzy neural network will generally obtain a fuzzy neural network model with good performance. The membership function adopted is shown in Eq. (1).

1 $$u_{ij} = \exp \left( { - \frac{{\left( {x_{i} - m_{ij} } \right)}}{{\sigma_{ij}^{2} }}} \right)$$

where *u*_*ij*_ is the fuzzy membership value of the *j*-th node corresponding to the *i*-th variable; *x*_*i*_ is the *i*-th input variable; *m*_*ij*_ is the membership function cluster center of the *j*-th node corresponding to the *i*-th variable; *σ*_*ij*_ is the width of the membership functions the *j*-th node corresponding to the *i*-th variable.

The defuzzification function of the network activation layer is the sum of the products of the inputs of each node in the fuzzy layer, as shown in Eqs. (2) and (3). The defuzzification function is an improvement point in addition to the improvement of network structure, because the defuzzification function form of the general fuzzy neural network is similar to Eq. (4), there is a complex calculation problem. Therefore, in order to reduce the calculation difficulty and realize fast calculation, the network has improved its network defuzzification function.

2 $$y_{i} = \sum\limits_{i}^{m} {w_{i} \psi_{i} }$$

3 $$\psi_{i} = \prod\limits_{i = 1}^{n} {u_{ij} }$$

4 $$y_{i} = \frac{{\sum\nolimits_{i}^{m} {w_{i} \psi_{i} } }}{{\sum\nolimits_{i}^{m} {\psi_{i} } }}$$

where *w*_*i*_ is the weight of the *i*-th node in the reasoning layer; ψ_*i*_ is the algebraic product of the membership degree of the *i*-th node in the reasoning layer; *u*_*ij*_ is the *i*-th variable corresponds to the membership degree of the *j*-th node; *n* is the number of variables; *m* is the number of reasoning layer nodes.

The essence of classification evaluation of top coal cavability is a multi-classification problem, and the softmax function is a typical activation function in the neural network of multi-classification problems and a very mature activation function^19^. Therefore, the softmax function activates the clear value after defuzzification, and then the final prediction value is obtained. The softmax function is shown in Eq. (5).

5 $$y_{t,k} = \frac{{e^{{\left( {y_{t,k} } \right)}} }}{{\sum {_{k} e^{{\left( {y_{t,k} } \right)}} } }}$$

where *y*_*t,k*_ is the *t*-th sample is the output value of the *k*-th neuron of the activation layer.

The softmax cross-entropy loss function can measure the similarity between the predicted and actual values in the neural network of multi-classification problems^20^. Therefore, the softmax cross-entropy loss function is used as the loss function of this paper. Softmax cross-entropy loss function, as shown in Eq. (6).

6 $$L\left( {\hat{y},y^{*} } \right) = - \sum\limits_{i}^{Nclass} {y_{i}^{*} \ln \left( {\hat{y}_{i} } \right)}$$

where $\hat{y}$ is the predicted label vector; y* is the actual label vector.

The reverse adjustment gradient of the network is shown in Eqs. (7)–(9):

7 $$\Delta m_{ij} = - \eta \frac{\partial L}{{\partial m_{ij} }} = - \eta \left( {y_{t,k} - y^{*} } \right)w_{ij} \prod\limits_{l = 1,l \ne i}^{n} {u_{li} \cdot 2\exp \left[ { - \left( {x_{i} - m_{ij} } \right)\sigma_{ij}^{ - 2} } \right]} \cdot \frac{{x_{i} - m_{ij} }}{{\sigma_{ij}^{2} }}$$

8 $$\Delta \sigma_{ij} = - \eta \frac{\partial L}{{\partial \sigma_{ij} }} = - \eta \left( {y_{t,k} - y^{*} } \right)w_{ij} \prod\limits_{l = 1,l \ne i}^{n} {u_{li} \cdot 2\exp \left[ { - \left( {x_{i} - m_{ij} } \right)\sigma_{ij}^{ - 2} } \right]} \cdot \frac{{\left( {x_{i} - m_{ij} } \right)^{2} }}{{\sigma_{ij}^{3} }}$$

9 $$\Delta w_{ij} = - \eta \frac{\partial L}{{\partial w_{ij} }} = - \eta \left( {y_{t,k} - y^{*} } \right)w_{ij} \psi_{i}$$

where *L* is the loss function; *y*_*t,k*_ is the *t*-th sample is the output value of the *k*-th neuron of the activation layer; y^*^ is actual value label vector; *η* is the learning rate; *u*_*ij*_ is the *i*-th variable corresponds to the membership degree of the *j*-th node; *x*_*i*_ is the *i*-th input variable; *m*_*ij*_ is the membership function cluster center of the *j*-th node corresponding to the *i*-th variable; *σ*_*ij*_ is the width of the membership functions the *j*-th node corresponding to the *i*-th variable; ψ_*i*_ is the algebraic product of the membership degree of the *i*-th node in the reasoning layer; *w*_*ij*_ is the weight; *n* is the number of variables.

### Verification of the global approximation of the improved fuzzy neural network

Both artificial and fuzzy neural networks are general approximators^21,22^, i.e., they both have global approximations. Therefore, it is necessary to demonstrate the global approximation of the proposed improved fuzzy neural network to prove that the modified network is still a neural network and possesses its properties. However, it is an effective method to use the Stone-Weirstrass theorem to prove the global approximation of the network^23^. Therefore, the Stone-Weirstrass theorem is used to demonstrate the global approximation of the improved fuzzy neural network. To prove the improved fuzzy neural network, only need to prove that the output function y satisfies the three lemmas in the Stone-Weirstrass theorem^24^:

1. (*y*, *d*_∞_) is an algebra, i.e., *y* is closed to addition, multiplication, and number multiplication.
  To prove that (*y*, *d*_∞_) is an algebra, assume that *y*_1_, *y*_2_ ∈ *y*.
  So
  10 $$y_{1} (x) = \sum\limits_{{i_{1} = 1}}^{{m_{1} }} {w_{{i_{1} }} } \prod\limits_{j = 1}^{n} {u_{{ji_{1} }} } \left( {x_{j} } \right)$$
  11 $$y_{2} (x) = \sum\limits_{{i_{2} = 1}}^{{m_{2} }} {w_{{i_{2} }} } \prod\limits_{j = 1}^{n} {u_{{ji_{2} }} } \left( {x_{j} } \right)$$
  Then
  12 $$\begin{aligned} y_{1} (x) \cdot y_{2} (x) & = \sum\limits_{{i_{1} = 1}}^{{m_{1} }} {w_{{i_{1} }} } \prod\limits_{j = 1}^{n} {u_{{ji_{1} }} } \left( {x_{j} } \right) \cdot \sum\limits_{{i_{2} = 1}}^{{m_{2} }} {w_{{i_{2} }} } \prod\limits_{j = 1}^{n} {u_{{ji_{2} }} } \left( {x_{j} } \right) \\ & = \sum\limits_{{i_{1} = 1}}^{{m_{1} }} {\sum\limits_{{i_{2} = 1}}^{{m_{2} }} {w_{{i_{1} }} } } \cdot w_{{i_{2} }} \left( {\prod\limits_{j = 1}^{n} {u_{{j_{1} }} } \left( {x_{j} } \right) \cdot u_{{j_{2} }} \left( {x_{j} } \right)} \right) \\ \end{aligned}$$
  Since both *u*_*ji*1_(*x*_*j*_)and *u*_*ji*2_(*x*_*j*_) are Gaussian, they are still Gaussian after being multiplied. Therefore, the above formula can be equivalent to Eq. (2), and *y*_1_(*x*)⋅*y*_2_(*x*) ∈ *y* can be proved.
  At the same time, for any *c* ∈ *R* it is easy to obtain:
  13 $$cy_{1} (x) = \sum\limits_{{i_{1} = 1}}^{{m_{1} }} c w_{{i_{1} }} \prod\limits_{j = 1}^{n} {u_{{ji_{1} }} } \left( {x_{j} } \right)$$
  It can be seen that the above formula is equivalent to Eq. (2), so *cy*_1_(*x*) ∈ *y*.
  Similarly,
  14 $$y_{1} (x) + y_{2} (x) = \sum\limits_{{i_{1} = 1}}^{{m_{1} }} {w_{{i_{1} }} } \prod\limits_{j = 1}^{n} {u_{{ji_{1} }} } \left( {x_{j} } \right) + \sum\limits_{{i_{2} = 1}}^{{m_{2} }} {w_{{i_{2} }} } \prod\limits_{j = 1}^{n} {u_{{ji_{2} }} } \left( {x_{j} } \right)$$
  It can be seen that the above formula is equivalent to Eq. (2), so *y*_1_(*x*) + *y*_2_(*x*) ∈ *y*.
  Based on the above, it can be proved that (*y*, *d*_∞_) is an algebra.
2. (*y*, *d*_∞_) can be separated on U, i.e., for any given *x*^0^, *y*^0^ ∈ U, when *x*^0^ ≠ *y*^0^, *y*(*x*^0^) ≠ *y*(*y*^0^).

In order to prove that (*y*, *d*_∞_) can be separated on U, suppose that *x*^0^  = (*x*_1_^0^,* x*_2_^0^,* x*_2_^0^, … , *x*_n_^0^), *y*^0^ = (*y*_1_^0^, *y*_2_^0^, *y*_2_^0^, … , *y*_n_^0^), if *x*_*i*_^0^ ≠ * y*_*i*_^0^, define two fuzzy sets (*A*_*i*_^1^, *u*_*i*_^1^) and (*A*_*i*_^2^, *u*_*i*_^2^) on the *i*-th subspace on U, and the corresponding membership function are :

15 $$u_{{A_{i}^{1} }} \left( {x_{i} } \right) = \exp \left( { - \frac{{\left( {x_{i} - x_{i}^{0} } \right)^{2} }}{2}} \right)$$

16 $$u_{{A_{i}^{2} }} \left( {x_{i} } \right) = \exp \left( { - \frac{{\left( {x_{i} - y_{i}^{0} } \right)^{2} }}{2}} \right)$$

At the same time, two fuzzy sets (*B*^1^, *u*_*B*1_^1^) and (*B*^2^, *u*_*B*2_^1^), are defined on the output universe R, and the corresponding membership functions are:

17 $$u_{B}^{i} (w) = \exp \left( { - \frac{{\left( {w - w_{j} } \right)^{2} }}{2}} \right)\quad j = 1,2$$

In addition, suppose that the fuzzy rule base is composed of two fuzzy rules, i.e., *m* = 2.

Based on the above assumptions:

18 $$y\left( {x^{0} } \right) = w_{1} + w_{2} \prod\limits_{i = 1}^{n} {\exp } \left( { - \frac{{\left( {x_{i}^{0} - y_{i}^{0} } \right)^{2} }}{2}} \right)$$

19 $$y\left( {y^{0} } \right) = w_{2} + w_{1} \prod\limits_{i = 1}^{n} {\exp } \left( { - \frac{{\left( {x_{i}^{0} - y_{i}^{0} } \right)^{2} }}{2}} \right)$$

20 $$\prod\limits_{i = 1}^{n} {\exp \left( { - \frac{{\left( {x_{i}^{0} - y_{i}^{0} } \right)^{2} }}{2}} \right)} = 1$$

Since $x_{i}^{0} \ne y_{i}^{0}$ has been assumed, there is no situation of Eq. (20). At this time, when *w*_1_ ≠ *w*_2_, there must be *y*(*x*^0^) ≠ *y*(*y*^0^). It can be proved that (*y*, *d*_∞_) can be separated on U.

(3) All points on (*y*, *d*_∞_) are not 0, i.e., every *x*_*i*_ ∈ U has *y*( *x*_*i*_ ) ∈ U, so that *y*(*x*) ≠ 0.

From Eq. (2), know that *w*_*i*_ ≠ 0 (*i* = 1, 2, 3, …, m) and the obtained *y* are not zero. Therefore, all points on the (*y*, *d*_∞_) are not 0.

The above proof shows that the improved fuzzy neural network satisfies the three lemmas in the Stone-Weirstrass theorem, i.e., the improved fuzzy neural network has a global approximation. At the same time, it shows that the improved fuzzy neural network is still a neural network and has the properties of a neural network.

## Influencing factors of top coal cavability and its evaluation grade division

### Influencing factors of top coal cavability

The two main factors that affect the top coal cavability are geological factors and mining technology factors. Between them, geological factors occupy a dominant position. Because geological factors often determine the technical means that should be adopted in longwall top coal caving mining^25^. Therefore, this paper mainly studies the top coal cavability under geological factors. According to engineering practice, the burial depth of the coal seam (H) the thickness of the coal seam (M), the thickness of the gangue (m_j_), the uniaxial compressive strength of the coal (Rc), the degree of crack development (DN, i.e., the product of the number of through fractures N on the coal surface of 1 m^2^ and the fractal dimension D_1_ of the distribution of the number of fractures counted by the coal specimen), and the filling coefficient of the direct roof (*k*, *k* = Σ*hk*_*p*_/*M*) are important geological factors that affect the top coal cavability^26^. Therefore, this paper takes the factors mentioned above as the influencing factors for evaluating top coal cavability.

### Classification of top coal cavability

The classification of top coal caving properties is itself a problem of fuzziness, complexity, and uncertainty. However, this paper aims to verify the applicability and superiority of the improved and optimized fuzzy neural network in the classification evaluation of top coal cavability. Therefore, this paper did not discuss classification standards and methods of top coal cavability. Therefore, it is only classified into I II, III, and IV based on the broad experience of field workers. The specific conditions of each level are shown in Table 1.

**Table 1:** Classification of top coal cavability.

Grade	Cavability	Description
I	Excellent	The top coal can cave well. As long as the appropriate coal caving support is selected, the coal can be discharged without additional measures
II	Good	The top coal can also cave well. Similarly, the coal can be discharged after selecting a suitable coal caving support. However, there are large pieces of coal in the discharged coal, which is prone to blockage, and corresponding measures need to be taken
III	Medium	The top coal can cave, but it does not cave very well. At the same time, there are large blocks of discharged coal and blockage often occurs. Corresponding measures must be taken to discharge the coal
IV	Poor	The top coal caving is extremely difficult, and more measures are needed to discharge the coal barely

## Data and data preprocessing

Since the sample data is not easy to obtain, the sample data in this paper is obtained from the database of published papers from CNKI. The obtained sample data are 61 groups. However, some samples in the obtained sample data have missing parameters. This paper uses the average value within the grade to fill the missing parameters for the data with missing parameters considering the scarcity of sample data. At the same time, to minimize the influence of outliers on the model, the Mahalanobis distance method^27^ is used to detect outliers, and samples detected as outliers at a confidence level of 0.5% are eliminated. After processing, 60 groups of data samples are obtained, as shown in attached Table 2.

**Table 2:** Sample data.

Number	Working face	H (m)	Rc (MPa)	m_j_ (m)	M (m)	DN	k	Grade
1	Micun Mine 21	300	14	0	8	10.05	1	II
2	Zhangshuanglou Mine	600	14.6	0.3	4.5	18	1.25	II
3	Xinzhuang Mine 3#	175	10	0	7	13.02	0.78	II
4	Dongliang Mine 2–4#	300	16	0.3	12.5	14.8	1.14	III
5	Ciyaogou Mine 11,101	201	28.53	0.41	4.93	1.44	1.44	IV
6	Xuecun 2#	300	16	0.4	14.5	15.2	0.36	III
7	Yaoqiao 3#	193	25	0.3	6.7	7.5	0.71	IV
8	Pingdingshan Mine E-15#	300	14	0	7.9	12.09	1.16	II
9	Shanxi X Coal Mine 8301–5#	484	24	0.26	6.39	9	1.4	IV
10	Jiaojiazhai 5#	140	2.8	0	6.5	14.71	1.2	II
11	Shanxi X Mine 8101–8 + 10#	273	13.8	0.3	9	12	2.3	III
12	Nantun Mine 5#	400	15	0.2	3.2	10.22	0.78	II
13	Xiangshan Mine 5#	230	8.5	0	5.5	16.08	0.72	II
14	Mengying Mine 15#	220	8.5	0	6.3	10.65	0.82	I
15	Xieqiao 13–1#	357	10	0.12	12	14	2.44	II
16	Xinglongzhuang Mine 3#	413	13.74	0.3	7.8	12.88	0.58	II
17	Singareni Coal Mine 3#	350	25.5	0.1	7	10.22	0.88	II
18	Liujialiang Mine 5#	140	2.8	0	6	19.4	1.2	I
19	Madigou Mine 13#	117	15.78	0.6	14.79	10.35	1.04	III
20	Lingwu Mine 2#	200	15	0	10.28	8.84	0.83	IV
21	Wangzhuang Mine	250	25	0.2	6.5	2	1.28	II
22	Shanxi X Mine 8101–5#	240	13.8	0.45	7.43	12.4	0.61	III
23	Dalong Mine 5#	160	5	0	8.4	16	0.73	II
24	Yuanzigou Mine 1,012,001	780	18	0.2	8.25	10.22	0.94	II
25	Gaozhuang Mine 4–17#	240	20	0.2	5.5	8.6	0.4	II
26	Puhe Mine (Lignite)	357	10	0.12	12	14	2.44	II
27	Changcun Mine	350	20	0.3	6.7	2	1.23	II
28	Anlin Coal Mine 28,071–2#	365	13.58	0	5.06	3	2.6	III
29	Baodian Mine 3#	435	16.6	0	8.8	9.23	0.52	II
30	Tangan Mine 3#	200	15	0.3	6.5	13.82	0.81	II
31	Xingtai Mine 2#	360	20	0.4	6.2	8.3	0.41	III
32	Phoenix Mountain Mine 3#	140	35	0.1	5.5	8.01	0.62	IV
33	Yangquan Fourth Mine 15#	193	25	0.1	6.7	7.5	0.71	III
34	Purcell Fourth Mine B901	230	19	0.27	9.74	10.35	1.04	III
35	Shuiyu Mine 10#	190	6.5	0	7.2	15.77	1.44	I
36	Nanshan Mine 18#	150	10	0.4	12.2	11.2	1.34	III
37	Kangjiatan Mine 88,203–8#	264	20	0.15	7.46	10.35	1.04	III
38	Yangquan No.1 Mine 15#	250	20	0.3	6	8.01	1.91	III
39	Cuijiagou 4–2#	262	17.5	0.05	5.85	9.5	1.78	II
40	Dayan Second Mine 3#	435	16.6	0	8.8	9.23	0.52	II
41	Xinzhouyao Mine 11#	300	30	0.4	8.6	9.85	0.25	IV
42	Wangpo Coal 3208–3#	650	18	0	5.1	10.22	0.94	II
43	Xinglongcun 4316	357	13.34	0.05	8.11	10.22	0.41	II
44	Tao Second Mine 2#	290	6.5	0.2	7.2	15.77	1.44	I
45	Wulong Mine 214#	300	16	0.4	14.5	15.2	0.36	III
46	Gucheng Coal Mine 3#	610	10.66	0.17	6.33	10.22	0.94	II
47	Xiashi Festival 4–2#	177	17.5	0	12	9.1	0.17	III
48	Chaohua Coal Mine 2#	413	13.74	0.3	7.8	12.88	0.58	II
49	Dayang Coal Mine 3404–3#	432	10.08	0.58	5.72	1.04	0.94	II
50	Longgu Coal Mine 7201	580	15.2	0.17	6	15.77	1.1	I
51	Panjin Coal Mine 2301	220	13.3	0.6	17.03	10.22	0.94	II
52	Jialequan Mine 2–4#	128	10	0.52	8.2	9.11	0.56	IV
53	Gongwusu Coal Mine 1604	286	9.5	0.58	8.5	10.22	1.23	II
54	Zhaozhuang 2–1304#	440	14.03	0.19	5.36	11.98	0.44	II
55	Xinji Mine 13#	450	8	0.3	7.2	15.77	0.43	I
56	Yongan Mine 3#	200	5.7	0.2	6.5	17	0.6	IV
57	Wangjiazhuang Mine 3#	200	16	0	7	7.43	0.56	III
58	Zhangcun Mine	230	20	0.2	7	2	0.68	II
59	Hemei No.3 Mine	750	16.5	0.3	8	10.22	0.33	II
60	Xuzhou Qishan 3#	300	14	0	8	10.1	1	II

*Data comes from the database of published papers on CNKI.

## Fuzzy neural network design and its training and test

### Fuzzy neural network design

The network design generally consists of several parts: the network layer number design, the neuron number setting of each layer in the network, the over-fitting prevention design, the optimization design to improve network convergence speed, the initial value setting, and other hyperparametric settings. Therefore, according to the needs of the classification evaluation top coal cavability and the characteristics of the data obtained, this paper is designed according to the improved fuzzy neural network principle.

1. Network layers design

The number of network layers is closely related to the prediction output error. It can be proved from theory and practice that the prediction output error will continue to decrease with the increase of network layers. Nevertheless, while increasing the number of network layers, the neural network model will also become more complex, increasing the training time. Studies^28^ have shown that increasing the number of neurons in the hidden layer to improve training accuracy is easier to achieve a good training effect than increasing the number of network layers to improve training accuracy, and at the same time, it can reduce training time to a certain extent. This paper hopes to get a better training effect while spending less training time. Therefore, the neural network designed is a simple fuzzy neural network with five layers: the input layer, membership function generation layer, inference layer, activation layer, and output layer.

(2) Setting the number of neurons in each layer of the network

The geological factors affecting the top coal cavability main include burial depth, coal seam thickness, gangue thickness, uniaxial compressive strength of coal, fracture development degree, and direct top coal filling coefficient. The data parameters obtained are also mainly the above parameter data. Therefore, there are six neurons in the input layer. The membership function generation layer and reasoning layer belong to the hidden layer. Studies have shown that increasing the number of hidden neurons can effectively reduce the training error, but it does not reduce the error indefinitely. When the number of neurons in the hidden layer reaches a specific value, the error will not only not decrease. On the contrary, the error will increase, and the network will also lose its generalization ability. However, if the number of neurons in the hidden layer is too small, the training error cannot be reduced. Therefore, according to the empirical Eq. (21)^29^, this paper determines that the number of neurons in the membership generation layer is 18, and the number of neurons in the reasoning layer is 6. In this paper, the grade of top coal cavability is 4, so the number of neurons in the activation layer is set to 4. In addition, this paper only needs to output a parameter (top coal cavability grade), so the network output layer neuron is 1.

21 $$N_{h} = 2N - 1$$

where *N*_*h*_ is the number of hidden layer neurons; *N* is the number of neurons in the input layer.

(3) Anti-overfitting design and accelerating network convergence design

Neural networks generally encounter two problems, "overfitting" and slow network convergence during the training process. Therefore, corresponding measures shall be taken for this problem. For the "overfitting" problem, generally using regularization to optimize the network can have a good effect. For slow network convergence, using variable learning rate and Adam (Adaptive Motion Estimate) optimization algorithm to optimize the network design further will generally achieve good results and significantly improve the network's convergence speed^30^. Therefore, this paper adopts regularized optimization design to prevent the overfitting problem in the training process; Adam optimization algorithm and a variable learning rate with exponentially decayed learning rate are used to achieve rapid network convergence. Then add a regular term after the loss function, as shown in Eq. (22); The network reverses the adjustment gradient, and the Adam algorithm flow are shown in Eqs. (23)–(33).

22 $$L = - \sum\limits_{i}^{Nclass} {y_{i}^{*} \ln \left( {\hat{y}_{i} } \right)} + \frac{\lambda }{2}\sum\limits_{j = 1}^{m} {w_{ij}^{2} }$$

The network reverses adjustment gradients are:

23 $$\eta_{t} = \eta_{0} \cdot \beta^{t} + \varpi$$

24 $$\Delta w_{ij} = - \eta_{t} \frac{\partial L}{{\partial w_{ij} }} = - \eta_{t} \left[ {\left( {y_{t,k} - y^{*} } \right)w_{ij} \psi_{i} + \frac{\lambda }{n}w_{ij} } \right]$$

25 $$\Delta m_{ij} = - \eta_{t} \frac{\partial L}{{\partial m_{ij} }} = - \eta_{t} \left( {y_{t,k} - y^{*} } \right)w_{ij} \cdot \prod\limits_{l = 1,l \ne i}^{n} {u_{li} } \cdot 2\exp \left( { - \frac{{\left( {x_{i} - m_{ij} } \right)}}{{\sigma_{ij}^{2} }}} \right)\frac{{\left( {x_{i} - m_{ij} } \right)}}{{\sigma_{ij}^{2} }}$$

26 $$\Delta \sigma_{ij} = - \eta_{t} \cdot \frac{\partial L}{{\partial \sigma_{ij} }} = - \eta_{t} \left( {y_{t,k} - y^{*} } \right)w_{ij} \cdot \prod\limits_{l = 1,l \ne i}^{n} {u_{li} } \cdot 2\exp \left( { - \frac{{\left( {x_{i} - m_{ij} } \right)}}{{\sigma_{ij}^{2} }}} \right)\frac{{\left( {x_{i} - m_{ij} } \right)^{2} }}{{\sigma_{ij}^{3} }}$$

27 $$V_{{\Delta w_{ij} }} = \kappa_{1} V_{{\Delta w_{ij} }} + \left( {1 - \kappa_{1} } \right) \cdot \Delta w_{ij}$$

28 $$S_{{\Delta w_{ij} }} = \kappa_{2} S_{{\Delta w_{ij} }} + \left( {1 - \kappa_{2} } \right) \cdot \Delta w_{ij}^{2}$$

29 $$V_{{\Delta w_{ij} }}^{corrected} = \frac{{V_{{\Delta w_{ij} }} }}{{1 - \kappa_{1}^{t} }}$$

30 $$S_{{\Delta w_{ij} }}^{corrected} = \frac{{S_{{\Delta w_{ij} }} }}{{1 - \kappa_{2}^{t} }}$$

31 $$\Delta w^{\prime}_{ij} = - \alpha \frac{{V_{{\Delta w_{ij} }}^{corrected} }}{{\sqrt {S_{{\Delta w_{ij} }}^{corrected} + \varepsilon } }}$$

Similarly,

32 $$\Delta m^{\prime}_{ij} = - \alpha \frac{{V_{{\Delta m_{ij} }}^{corrected} }}{{\sqrt {S_{{\Delta m_{ij} }}^{corrected} + \varepsilon } }}$$

33 $$\Delta \sigma^{\prime}_{ij} = - \alpha \frac{{V_{{\Delta \sigma_{ij} }}^{corrected} }}{{\sqrt {S_{{\Delta \sigma_{ij} }}^{corrected} + \varepsilon } }}$$

where Δ*w*_*ij*_, Δ*m*_*ij*_, and Δ*σ*_*ij*_ are network initial adjustment gradients; *n* is the total number of samples; *η*_0_ is the initial learning rate; *η*_*t*_ is the real-time learning rate; *t* is the number of iterations; *β* is the decay index, which is 0.9; ϖ is the constan to prevent the learning rate from returning to 0, which is 10^–5^; λ is the regular parameter; *κ*_1_, *κ*_2_, *ε*, *α* are the hyperparameters, among which is 0.9, 0.99, 10^–8^, and 0.001; *V*_∆__*wij*_, *V*_∆__*mij*_, *V*_∆__*σij*_ are intermediate amounts, the initial values are 0; *V*_∆*wij*_^*Corrected*^, *V*_∆*mij*_^*Corrected*^, *V*_∆*σij*_^*Corrected*^ are correction amounts; ∆*w*′_*ij*_, ∆*m*′_*ij*_, ∆*σ*′_*ij*_ are the the network finally adjusts the gradients.

(4) Initial weight and other initial value settings

The parameters in the membership function of the improved fuzzy neural network mainly include the membership function's cluster center (*m*) and the membership function's width (*σ*), and it is also the first step to generate the membership in the network. In addition, the membership function's cluster center and the membership function's width will be adjusted accordingly according to the error. Therefore, according to the number of neurons in the input and membership generation layers, the initial membership function cluster center and the membership function width are both 6 × 18 random Gaussian distribution matrix with a value range of [0,1].

The network designed in this paper does not have weight transfer in the input layer, membership function generation layer, and fuzzy layer, so it only needs to set the weight from the fuzzy layer to the active layer and set the initial weight as an 18 × 4 random Gaussian matrix with the value range of [0,1].

Since this paper adopts a variable learning rate that decays exponentially, the initial learning rate of the network can take an immense value, so the initial learning rate in this paper is set to 3.0.

The setting of the maximum number of training iterations will directly affect the generalization ability of the network. Generally, with the increase of the number of training iterations, the training error and test error will decrease, but with the increase of the number of iterations, the phenomenon of "overfitting" will appear, resulting in the increase of test error instead of decreasing. For this reason, since the network is not complex, the number of iterations is set to 100.

When the training reaches a specific error requirement, the operation should be stopped, which can prevent overfitting to a certain extent and effectively reduce the training time. Generally, the training error can meet the requirements when it reaches 10^−4^. Therefore, this paper sets the training error stops as 10^−4^.

(5) Network training and test design

**Figure 3:**
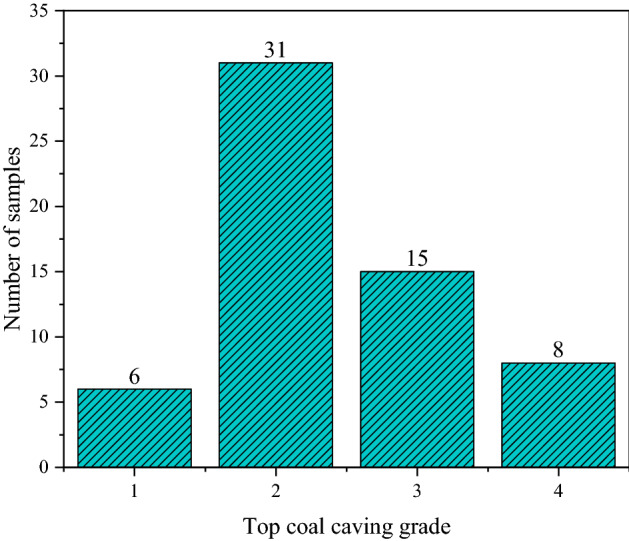
Top coal cavability grade distribution.

Network training and testing require at least one test set and one test set, and the two sets are required to be independent of each other. Since there are only 60 groups of samples after data preprocessing, this paper takes the sample number of 1–50 as the training sample and the sample number of 51–60 as the test data. In addition, the model's generalization ability is an important parameter to measure the reliability and robustness of the model. However, if the model's generalization ability can be verified during training, the model's generalization ability can be well measured to a certain extent, and the overfitting problem can be avoided to a certain extent. Therefore, the K-fold cross-validation method is used to train the model, and part of the training set is divided into the training set, and the other part is the validation set. Due to the small number of samples, to better test the model's generalization ability, the training adopts the tenfold cross-validation method to train the model. In addition, it is found from the sample data that there is an imbalance in the grade of top coal cavability, so this issue needs to be considered in order to obtain a stable and robust prediction model. Stratified sampling can alleviate the imbalance of sample categories to a certain extent, so the tenfold cross-validation of stratified sampling is used to train the model. The top coal cavability grade distribution is shown in Fig. 3.

### Analysis of network training and test results

**Figure 4:**
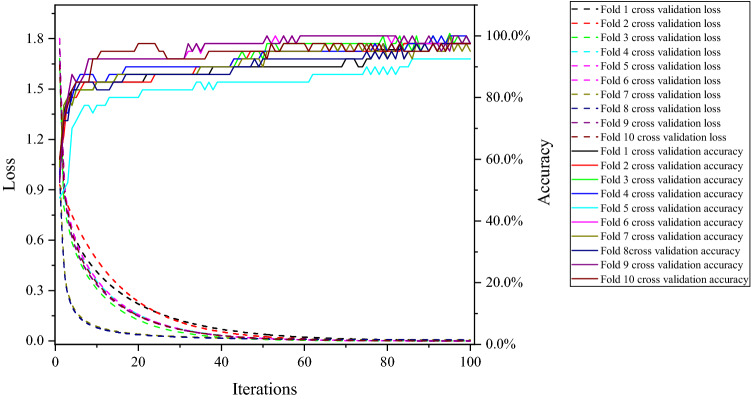
The training chart of tenfold cross-validation.

**Figure 5:**
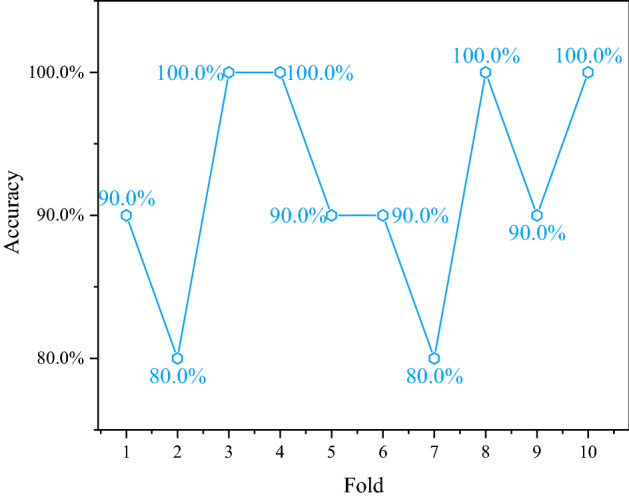
The validation chart of tenfold cross-validatio.

**Figure 6:**
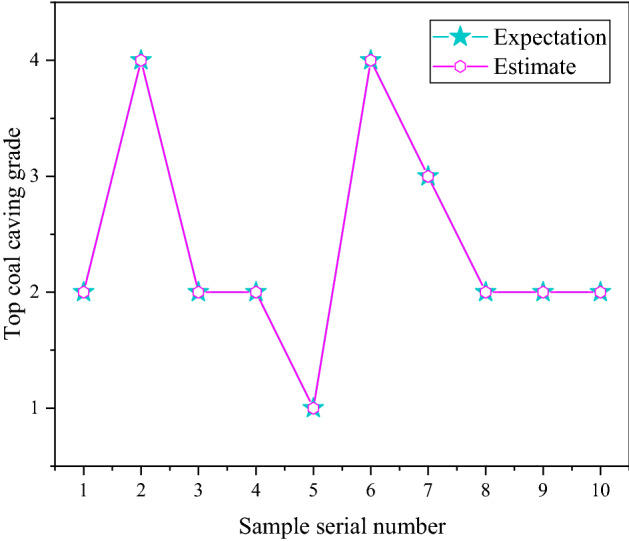
Model test results.

After the above design, MATLAB was used to implement it, and it was trained and tested. Finally, the training diagram of the tenfold cross-validation (as shown in Fig. 4), the validation diagram of the tenfold cross-validation (as shown in Fig. 5), and the test result graph (shown in Fig. 6) are obtained. Through the tenfold cross-validation training chart in Fig. 4, it can be seen that in each fold cross-validation, the prediction accuracy of the network is as expected, and the accuracy increases with the increasing number of iterations. At the same time, when the model is fully converged, the prediction accuracy rate of the model reaches more than 92.5%, i.e., the number of prediction correct samples reaches 37 or more in the 40 training samples of the model. It shows that the model can fit the benchmark data well during training. From the prediction accuracy of the verification set in each fold in Fig. 5, the verification prediction accuracy of each fold in the training process reaches more than 80%, i.e., among the 10 verification samples, more than 8 samples were predicted correctly by the model. In addition, the total verification average prediction rate of tenfold cross-validation reached 92%. It shows that the model has good generalization ability, and there is no phenomenon such as overfitting and underfitting. However, this generalization ability has also been well verified from the final test. In the test stage, the 10 samples used for the test are correctly predicted, i.e., the positive prediction rate of the network test is 100%. The above training, verification, and testing fully show that the optimized and improved network has good generalization and is suitable for evaluating top coal cavability.

## Engineering practical application

The established fuzzy neural network prediction model is applied to the top coal cavability grade evaluation of 61,107 Longwall top coal caving working face in Liuwan Coal Mine. Liuwan Coal Mine is subordinate to Shanxi Fenxi Mining (Group) Co., Ltd., located in Luliang City, Shanxi Province, China. The coal seam in the 61,107 longwall top coal caving working face of Liuwan Coal Mine is No. 11 coal seam. The average buried depth of No. 11 coal seam is 240 m. The average coal thickness is about 4.35 m. There is a layer of gangue in the coal seam, and the thickness of gangue is about 0.2 m. The roof of the coal seam can be mined with caving, and the filling coefficient of the direct roof is 0.77. The coal in the coal seam has relatively developed cracks (DN = 12.5). In addition, the coal quality is better, with the characteristics of metallic luster and high hardness. The compressive strength is about 12.5 MPa.

Through the model prediction and evaluation, the prediction and evaluation result is that the top coal cavability grade of 61,107 longwall top coal caving face in Liuwan Coal Mine is grade II. From the project site, the predicted results are consistent with the actual site. In the production process, the 61,107 longwall top coal caving working face can cave well and be well discharged from the coal caving support, but there are large blocks in the discharged coal and blocking occasionally. It can be well solved by taking corresponding measures.

## Conclusion

The fuzzy neural network developed by Nauck and Kruse is improved and optimized because of the shortcomings of the current classification evaluation of top coal cavability. Moreover, the global approximation of the improved neural network that the neural network should have is demonstrated. In order to avoid the "overfitting" problem of the model, improve the rapid convergence of the neural network model and make it have good generalization ability, the corresponding optimization design has been made. The model was constructed using MATLAB software and was trained and tested. Finally, the trained and tested network evaluated the top coal cavability grade of the 61,107 longwall top coal caving working face of Liuwan Coal Mine. The prediction evaluation result was consistent with the actual situation of the project. It fully proves that the improved and optimized fuzzy neural network has good universal applicability in the classification evaluation of top coal cavability. It provides another more scientific and reliable method for the classification evaluation of top coal cavability.
